# Infants Distinguish Antisocial Actions Directed towards Fair and Unfair Agents

**DOI:** 10.1371/journal.pone.0110553

**Published:** 2014-10-16

**Authors:** Marek Meristo, Luca Surian

**Affiliations:** 1 Department of Psychology, University of Gothenburg, Göteborg, Sweden; 2 Department of Psychology and Cognitive Sciences, University of Trento, Rovereto (Trento), Italy; George Mason University/Krasnow Institute for Advanced Study, United States of America

## Abstract

Three experiments provide evidence of an incipient sense of fairness in preverbal infants. Ten-month-old infants were shown cartoon videos with two agents, the ‘donors’, who distributed resources to two identical recipients. One donor always distributed the goods equally, while the other performed unequal distributions by giving everything to one recipient. In the test phase, a third agent hit or took resources away from either the fair or the unfair donor. We found that infants looked longer when the antisocial actions were directed towards the unfair rather than the fair donor. These findings support the view that infants are able to evaluate agents based on their distributive actions and suggest that the foundations of human socio-moral competence are acquired independently of parental feedback and linguistic experience.

## Introduction

How do people acquire the ability to judge the fairness of resource distributions? What is the nature of the learning mechanisms involved in the development of this fundamental aspect of the human social brain? Several theories in moral philosophy and psychology posit that we rely on domain general learning mechanisms, either associationist (e.g. [1], [2]) or constructivist in nature [3]–[5]. In contrast, nativist theories emphasise the role of domain-specific principles [6], [7] and acquisition mechanisms [8], [9].

For many decades, developmental research has been guided by theories in the first camp and most of the results appeared to provide strong support for the constructivist models. Several studies have reported a slow development in children's judgments, showing that preschoolers' distributions are mainly guided by self-interest and that they are equitable only towards friends [10]. Young school-aged children instead tend to prefer egalitarian distributions, and it is only at the end of primary school that children distribute goods also according to proportional principles that take into account the recipients' relative need and merit [11]–[16]. By the age of six years, their desire to be fair depends, similarly to adults, on a concern to appear fair to others and such concern increases in the following years [17], [18].

Recent research has found evidence that is inconsistent with the slow development predicted by the early constructivist accounts. Olson and Spelke [19] reported that preschoolers' distributions of resources are coherent with three principles discussed in depth in evolutionary biology: the principles of kin selection, direct reciprocity and indirect reciprocity. Namely, in their distributions children tend to favour agents who are similar to them (*kin selection*) and who have acted pro-socially towards them (*direct reciprocity*) or other people (*indirect reciprocity*). Therefore, preschoolers' judgments and distributions are not solely guided by self-interest (see also [20]). Baumard, Mascaro and Chevallier [21] found that while three-year-olds display a preference for egalitarian distributions, they are also able to take into account merit when forced to perform a non-egalitarian distribution of goods restricted by contextual factors, such as the scarcity of resources. Convergent evidence was reported by Kanngiesser and Warneken [22], who found that preschoolers took into account the relative amount of work done by the children themselves and by their collaborative partners when they had to share some rewards, and by Kenward and Dahl [23] who found that 4.5-year-olds, after watching agents behaving in helpful or hindering way toward another agent, gave more biscuits to the helper than to the hinderer.

Infant studies may pose an even stronger challenge to the theoretical accounts based on constructivist or associationist mechanisms. Four published studies have reported evidence suggesting that infants, in their second year of life, form egalitarian expectations about distribution of goods and evaluate egalitarian distributors more positively than non-egalitarian distributors. For example, by 15–19 months, infants prefer agents who have distributed goods equally compared to the agents who performed unequal distributions [24]; they look longer when goods are distributed unequally rather than equally among equal recipients [25], [26]; and they look longer when a third party approaches a fair rather than an unfair distributor [24]. Moreover, when the two potential recipients differ in the amount of work they have done, 21-month-old infants are surprised to see that goods are distributed equally between them, suggesting that they consider relative merit when forming expectations about resource distributions [26]. Overall, these studies suggest that a sense of fairness may already affect infants' socio-moral reasoning in their second year. In explaining these results, some authors have endorsed an evolutionary perspective, positing an evolved sense of fairness (24,26,27; see also [28]), whereas others have criticised such views and emphasised the possible effects of learning and experience, suggested by the apparent developmental changes occurring between the ages of 12 and 15 months [29].

To address this issue, it is potentially fruitful to further investigate the evaluation skills in preverbal infants, at an age in which it is less likely that expectations and evaluations of equal and unequal distributions may have emerged entirely due to the encoding of statistical regularities observed in the social environment. However, the mere fact that infants expect a certain type of distributions (say, equal) is not compelling evidence that they have a sense of fairness unless it is also shown that infants assign different values to different types of distributions.

One way to test whether such attribution of value takes place early in development is to look at infants' reactions to pro-social and antisocial actions, such as giving or taking away resources, directed towards agents who have previously performed a fair or an unfair distribution of resources. For example, in Meristo and Surian [27], infants saw two distributors giving away two strawberries to two identical recipients; a fair distributor gave one strawberry to each while the unfair distributor gave both strawberries to one recipient only and ignoring the other. During the following test event a new agent rewarded one of the distributors with a strawberry. Infants looked significantly longer when the pro-social action was directed towards the unfair distributor, compared to when the same action was directed towards the fair one, suggesting that they evaluated the fair distributor more positively than the unfair one. Hamlin, Wynn, Bloom and Mahajan [30] have followed a related strategy in studying how infants evaluate helping and hindering actions. They found that 8-month-olds preferred individuals who acted pro-socially towards helping agents and antisocially towards hindering agents, rather than individuals who acted pro-socially towards hinderers and antisocially towards helpers. If young infants look differently at anti-social actions directed towards fair and unfair distributors, this would provide further support for the hypothesis that they evaluate their distributive actions.

In the present study, infants saw two agents, the donors or distributors, performing either an equal or an unequal distribution of goods. Then, a third party either repeatedly hit and pushed away one of the two donors (Experiment 1), or took resources away from him (Experiment 2). If infants spontaneously link such anti-social actions to the previous distributive actions performed by the donors, they should look differently at the two test events.

## Experiment 1

### Method

#### Participants

Sixteen full-term healthy 10-month old infants participated (age range: 291 – 314 days; *M* = 303 days; *SD* = 8 days; 10 female, 6 male). Infants were recruited by contacting their parents who were randomly chosen from the birth register of the Swedish Tax Agency among families living in Gothenburg (Sweden). The parents were informed about the purpose and procedure of the study and gave signed consent. An additional 8 infants were tested, but excluded from the sample due to fussiness (3), technical problems (2) and because they did not look for at least 2.5 seconds to the test event (3). The Regional Gothenburg Ethical Review Board approved the study.

#### Procedure, stimuli and apparatus

Infants were tested in a dimly lit room while they were sitting on their parent's lap approximately 60 cm from an eye-tracker monitor. The testing sessions started with a five-point infant calibration procedure. After the calibration, all infants were shown four *Donor familiarisation* events followed by one *Test* event (for details about the eye-tracking procedure and apparatus see [31].

**Figure 1:**
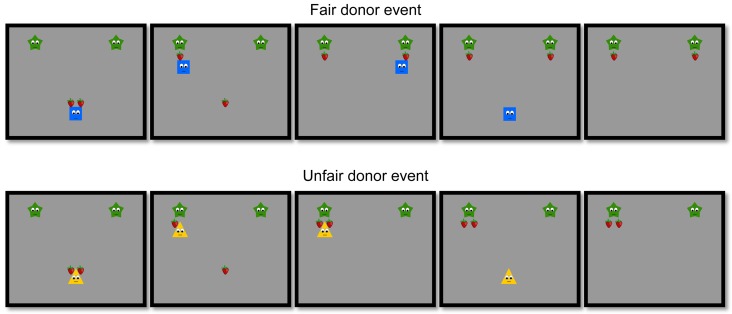
Selected frames from familiarization events shown to infants in Experiments 1 to 3. Each familiarization event started with the two stars present in the upper part of the screen. A donor entered with two strawberries and gave them to the stars. The donor performed either an equal distribution (the fair donor), giving one strawberry to each star, or an unequal distribution (the unfair donor), giving both strawberries to the same star. At the end of the distribution event the donor left the screen.

*Donor familiarisation phase:* During this phase infants saw two donors, depicted as a blue square and a yellow triangle with eyes and mouth, each performing two distributions towards two identical green stars, i.e. the recipients (Figure 1). Each distributive event started with the two stars present, one on the left side and one on the right side in the upper part of the screen. Then, one of the donors entered from the right or the left side carrying two red strawberries and gave them to the stars. Each donor performed either two fair (equal) distributions, giving one strawberry to each star, or two unfair (unequal) distributions, giving both strawberries to the same star. For half of the participants the order of the events in the donor familiarisation phase was Equal Unequal Unequal Equal, and for the other half the order was Unequal Equal Equal Unequal. Each time a star received a strawberry, it jumped twice. Information concerning the agency of the stars therefore included both morphological cues (eyes, mouth) and dynamic cues (autonomous motion, reaction-at-a-distance). At the end of each distribution event, the donors left the screen the same way they had entered and a new distributive event started.

**Figure 2:**
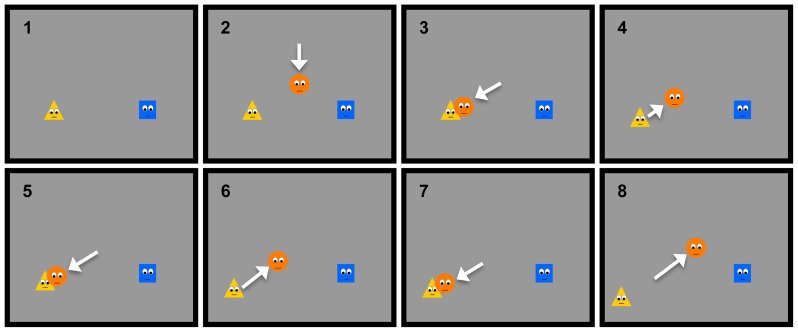
Selected frames from the test event used in Experiment 1. Infants first saw the two donors, familiar from the previous familiarization events, on the lower part of the screen. A new agent, an orange circle with eyes and mouth, entered from the middle of the upper part of the screen, moved down and stopped between the two donors for 0.5 seconds before it started to hit one of the two donors repeatedly for three times, each time pushing it farther away towards one of the lower corners. After the third hitting, the orange agent returned to the central position (arrows are inserted to illustrate the agent's motions).

*Test phase*: During this phase infants saw the two donors on the lower part of the screen. Then a new anti-social agent, an orange circle with eyes and mouth, entered from the middle of the upper part of the screen, moved down and stopped between the two donors for 0.5 seconds before it started to hit one of the two donors three times, each time pushing it farther away towards one of the lower corners (see Figure 2). After the third hitting, the agent returned to the central position, now closer to the other donor, and the scene froze for a maximum of 60 seconds.

The following variables were fully counterbalanced across the participants: (1) identity of the fair donor (yellow triangle vs. blue square), (2) order of donor familiarisation events (Equal Unequal Unequal Equal vs. Unequal Equal Equal Unequal), (3) side of the fair donor in the test event (Left vs. Right), and (4) type of test event (hitting the fair vs. hitting the unfair donor), resulting in 16 different testing sessions. The side of delivery of the first strawberry in both the equal and the unequal distribution events (Left vs. Right) co-varied with the identity of the fair donor (i.e. blue fair donors always delivered the first strawberry to the recipient on the left). All infants followed at least three donor familiarisation events.

The dependent measure was the time the infant spent looking at the still picture at the end of the test movie, from the moment when the anti-social agent finished the hitting (see Figure 1), until he or she looked away for at least 2.5 consecutive seconds, after having looked for at least 2.5 seconds. Preliminary analyses of the test event revealed no significant interaction of the test condition with the infant's gender in any of the three experiments.

### Results

**Figure 3:**
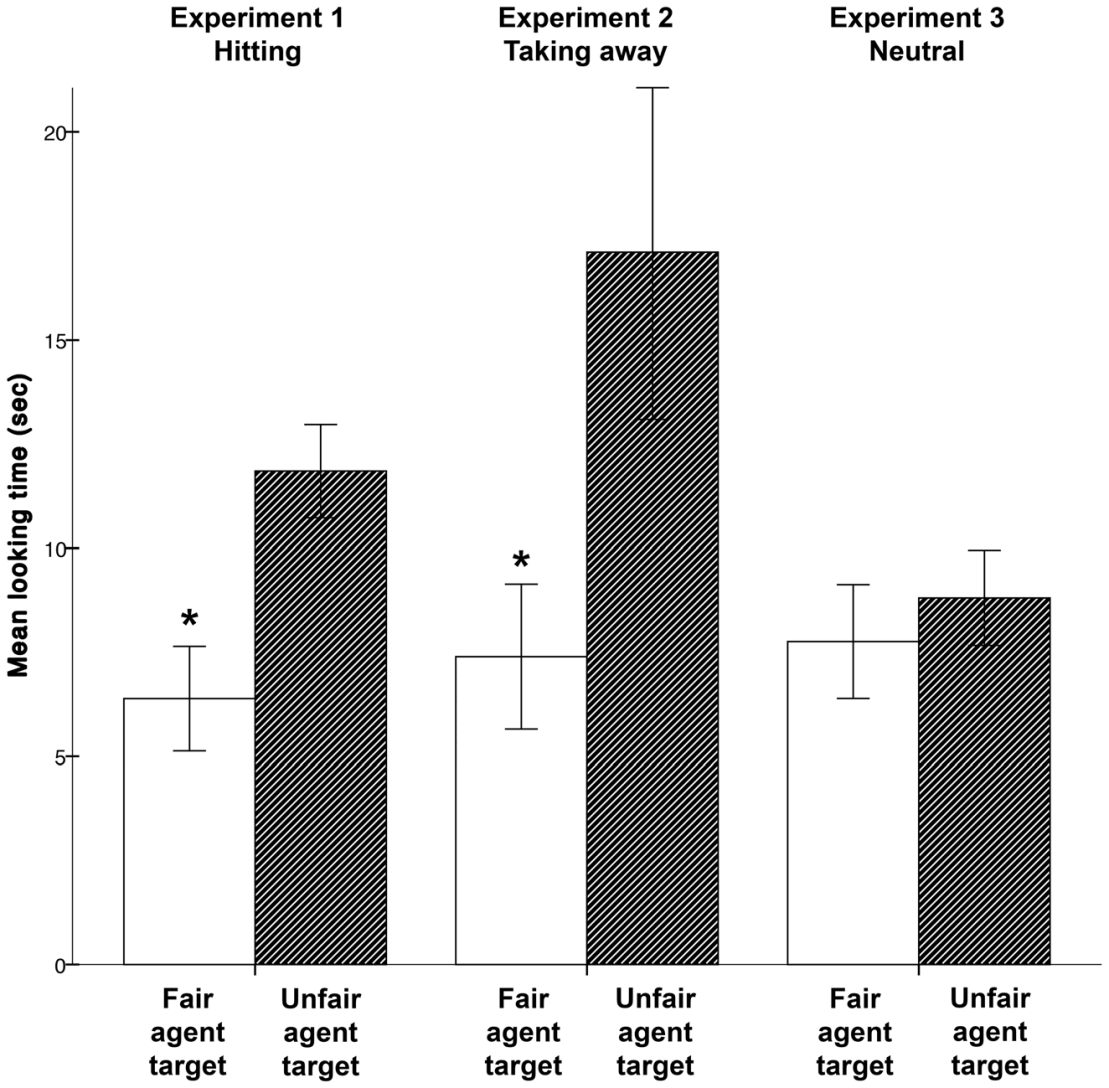
Mean looking times at the test events in Experiments 1 to 3.

Infants looked reliably longer at the test event involving the hitting of the unfair donor (*M* = 11.85 sec, *SD* = 3.17 sec) than at the event involving the hitting of the fair donor (*M* = 6.39 sec, *SD* = 3.55 sec), *t*(14) = 3.25, *p*  = .006, two-tailed, partial *η* ^2^  = .43, see Figure 3. However, the looking times were the opposite of what one would predict following the violation-of-expectation paradigm. That is, if infants expected the anti-social agent to act negatively towards the unfair donor rather than the fair one, and their reactions were mainly determined by noticing that one of the two test events violated such an expectation, they should have looked longer at the test event showing the fair agent being hit. Before discussing the possible explanations for these results, and their implications for current theories of early social-moral competence, we wanted to consolidate our findings in a new experiment showing a different kind of anti-social actions that involves taking away attractive resources (i.e. the anti-social action also used by Hamlin *et al.* [30].

## Experiment 2

The only difference between the present and the previous experiment was that in Experiment 2, the anti-social action shown in the test event consisted of taking away an attractive object from one of the two distributors.

### Method

#### Participants

A new group of 16 full-term healthy 10-month-old infants participated (age range: 294 – 313 days; *M* = 300 days; *SD* = 6 days; 8 female, 8 male). The recruitment procedure was the same as in Experiment 1. Seven additional infants were tested but excluded because they did not look for at least for 2.5 seconds at the test event (6), or because of fussiness (1).

#### Procedure and apparatus

**Figure 4:**
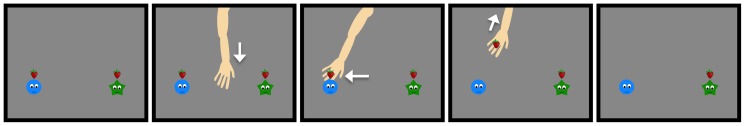
Selected frames from the test event used in Experiment 2. The test events began with the fair and unfair distributors present on the lower side of the screen. Both agents had one strawberry above and very close to them. An arm then came down from the upper side of the screen, stopped for 0.5 seconds in the middle of the screen between the two distributors and then approached one of them. The hand next took away the strawberry from one of the distributors and moved out of the scene, disappearing from the scene the same way it had entered.

The procedure and the apparatus were the same as in Experiment 1. Also, the familiarisation events were identical to the ones used in the previous experiment (see Figure 1; note that in Experiment 2, the two recipients in the donor familiarization events were yellow triangles, while the donors were a blue circle and a green star). The only difference concerned the test events. The test events began with both distributors present on the lower side of the screen and a drum sound to attract infants' attention to the screen. Both distributors had one strawberry above and very close to them (see Figure 4). Then an arm came down from the upper side of the screen, stopped for 0.5 seconds in the middle of the screen between the two distributors and then approached one of them. The hand next took away the strawberry from one of the distributors and moved out of the scene with it, disappearing from the scene the same way it had entered. The scene then froze to still, showing the two distributors, one with a strawberry above it and the other without.

### Results and Discussion

Infants looked reliably longer at the test event involving taking away the strawberry from the unfair donor (*M* = 17.11 sec, *SD* = 11.35 sec) than at the test event involving taking the strawberry away from the fair donor (*M* = 7.40 sec, *SD* = 4.92 sec), *t*(14) = 2.22, *p*  = .044, two-tailed, partial *η* ^2^  = .26 (Figure 3). In line with the results in Experiment 1, infants looked longer at the test event that showed the anti-social action directed towards the unfair donor.

We found in two experiments that infants looked reliably longer at test events showing an unfair donor being treated negatively by an anti-social agent compared to the test events showing a fair donor being treated the same way. This effect occurred both when the anti-social action involved hitting and pushing away (Experiment 1), and when it involved taking away an attractive object (Experiment 2). One possible explanation of these findings is that infants looked longer at events that they perceived as more coherent with the sequences of events seen in the previous familiarisation phase. However, in Meristo and Surian [27] it was found that infants looked longer when the reward was given to the unfair agent, rather than the fair one. This raises the possibility that infants, both in the previous study and in the present one, simply looked longer when an action was directed towards an unfair distributor, regardless of the negative or positive value of the action. This hypothesis was tested in the following experiment.

## Experiment 3

In Experiment 3, the procedure was similar to that of Experiments 1 and 2, except that the anti-social test event was replaced with a neutral action performed with an object that was very close to one of the donors. That object, instead of being taken away as in Experiment 2, was simply moved horizontally.

### Method

#### Participants

A new group of 16 full-term healthy 10-month-old infants participated (age range: 301 – 312 days; *M* = 305; *SD* = 3 days; 7 female, 9 male). The recruitment procedure was the same as in Experiments 1 and 2. Seven additional infants were tested but excluded because they did not look for at least for 2.5 seconds at the test event (1) or because of fussiness (6).

#### Procedure and apparatus

**Figure 5:**
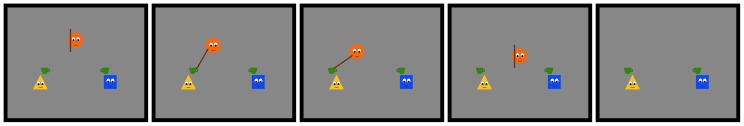
Selected frames from the test event used in Experiment 3. The event started with the fair and unfair distributors visible on the lower part of the screen. Above each of them there was a green cup. Shortly after the beginning of the trial an orange circle entered the scene from above and performing a neutral action with the cup that was either near the fair or the unfair agent. The action consisted in pushing the cup with a brown rod so that its final position was about 3 cm either to the right or to the left of its initial position, but not moving it closer or farther away from the donor.

The procedure, apparatus and familiarisation events were identical to Experiments 1 and 2. In the test events, the donors (i.e. a yellow triangle and a blue square) were visible on the lower part of the screen, one on the right side and the other on the left side (Figure 5). Above each donor there was a green cup. Shortly after the beginning of the trial, an orange circle (i.e. the agent that in Experiment 1 performed the anti-social action) entered the scene from above, and performed a neutral action with one of the two cups (i.e. the cup that was near the fair agent or the other cup that was close to the unfair agent). The action consisted in pushing the cup with a brown rod so that its final position was about 3 cm either to the right or to the left of its initial position, but not moving it closer or farther away from the donor. Counterbalancing factors included the identities of the fair and unfair donors (a yellow triangle or a blue square), the order of donor familiarisation events (Equal Unequal Unequal Equal vs. Unequal Equal Equal Unequal), the side of the fair donor in the test event (left or right), and the type of test event (moving the cup above the fair or the unfair donor). We measured the infants' looking times from the moment when the orange agent with the rod had moved out of the screen after having moved one of the cups, until the infants looked away for at least 2.5 consecutive seconds, after having looked for at least 2.5 seconds.

### Results

In contrast to the results in Experiments 1 and 2, infants were equally attentive to the test events involving actions directed towards the unfair donor (*M* = 8.81 sec, *SD* = 3.23 sec) and the actions directed towards the fair donor (*M* = 7.76 sec, *SD* = 3.87 sec), *t*(14)  = .59, *p*  = .57, two-tailed, partial *η* ^2^  = .03. An additional two 2 X 2 ANOVAs with Experiment as a one of the between-subject factors and Condition (fair vs. unfair donor targeted) as the second between-subject factor, yielded interaction effects that approach significance (Experiments 1 and 3: *F*(1,28) = 3.36, *p*  = .077 two-tailed, partial *η* ^2^  = .11; Experiments 2 and 3: *F*(1,28) = 3.24, *p*  = .082 two-tailed, partial *η* ^2^  = .10. This outcome provides no support for the claim that infants have a bias for looking at actions performed towards unfair donors and that such a bias can account for the results of Experiments 1 and 2 of the present study, as well as for the results reported by Meristo and Surian [27].

## General Discussion

The main finding of the present study is that infants looked longer at a test event when they saw an antisocial action performed towards an unfair rather than a fair donor (Experiments 1 and 2). This suggests that they preferred to look at the test events that were a coherent completion of the familiarisation events, displaying the donors' distributive actions, assuming that infants valued both the unfair distributions and the antisocial actions negatively. This conclusion is supported by Experiment 3, where the antisocial actions were replaced by a neutral object displacement action and where the infants looked equally long when such an action was performed with the object close to the fair or the unfair donor.

An alternative interpretation of our results is that, in our test situations, infants noticed that the third agent acting anti-socially in the test event was absent when the distributions happened previously, and therefore inferred that he did not know anything about the donors' fairness. Infants then looked longer at interactions with the unfair donor because of a general negativity bias; that is, a tendency to attend to potentially anti-social characters, demonstrated by Hamlin, Wynn and Bloom [32] with infants as young as three months old. Note that even this alternative interpretation implicates that infants are able to evaluate the donors on the basis of their distributions.

However, we found in Experiment 3 that infants did not look longer at the interactions with the unfair donor when the action directed towards it was neutral. Yet, the purpose of the action in Experiment 3 might not be easily encoded, which might have hindered the infants to generate any expectations, or their expectations might have been masked by their effort to understand the ambiguous action. On the other hand, the findings reported by Meristo and Surian [27] indicate that infants can overcome the negative bias. In Experiment 2 of that study, it was found that infants did not look longer at the interactions with the unfair donor when the third agent was hidden behind an opaque screen during the familiarisation phase and therefore was prevented from witnessing the donors' actions. In contrast, the ‘negative bias account' predicts that infants would attend longer to the interactions between the third party (acting pro-socially in that study) and the unfair donor, irrespective of the third party's informational state. While the ‘negativity bias’ can be ruled out for the case of events involving the prosocial actions investigated in Meristo and Surian [27], such alternative explanation cannot be conclusively rejected in the case of anti-social actions investigated in the present study and it would useful to test it directly in future.

Another possible explanation that needs to be discussed centres on infants' expectations about affiliative biases. When infants are facing a context in which a new third party is entering the scene, they generate expectations about its affiliative biases and look longer when the third party approaches an unfair rather than a fair distributor, provided that the purpose of the approach is easily encoded. When instead the purpose of the approach is not easily encoded, as it might have been the case in Experiment 3, infants do not generate any expectation, or their expectations are masked by their effort to understand the ambiguous action. Infants' affiliative biases towards one of the distributors are well documented in previous studies (e.g. [30], [33]) and this possibility is worth investigating in future studies.

Several studies on infants' socio-moral competence have found evidence consistent with the predictions generated following the violation of expectation paradigm. For example, Hamlin et al. [33] found longer looking times at test events showing a third party approaching a hinderer rather than a helper; Sloane et al. [26] found longer looks at unfair distributions between equal recipients; and Meristo and Surian [27] found that infants looked longer when an unfair agent, rather than a fair agent, received a reward. However, longer looking times at the test events that are instead coherent with the previous familiarisation events have also been reported in previous studies on infants' socio-moral reasoning [24], [34]. In the study by Kuhlmeier et al. [34], infants looked longer at test events showing a third party approaching a helper, rather than a hinderer, and Geraci and Surian [24] found that infants looked longer when they saw an agent standing next to a fair distributor rather than next to an unfair distributor. One possible explanation for these contrasting findings might be that in Kuhlmeier et al. [34] and Geraci and Surian [24], infants looked longer at scenes showing a third party approaching a helper/fair donor without any interaction following the approach. However, considering this variability in response, a complete account of these results clearly requires further studies aimed at identifying the factors that trigger a preference for events that violate expectations in some contexts, but yield the opposite preference for test events that are consistent with the information encoded during the familiarisation phase in other contexts.

Infants' preference for events showing anti-social actions directed towards unfair agents may be related to later biases of punishments directed towards deserving agents found in children and adults. Children and adults endorse the punishment of agents that are causally responsible for harm [3] and their motivation to punish unfair distributors is a consolidated finding in the literature in economic reasoning. Across a wide range of cultures, people asked to participate in the Ultimatum Game typically refuse offers received by distributors when such offers are judged to be unfair, that is inferior to the 20 % of the total good to be divided (e.g. [35]).

Free riders are also punished in other well-known tasks used to investigate economic reasoning, such as the Common Good Game [36]. Adults who care about the equality of distributions are more likely to punish free riders in the Common Good Game [37]. Also, neuroimaging studies have reported the selective activation of the anterior insula during such tasks, which indicates a crucial role of emotional reactions in the processes underlying moralistic punishment [38], [39].

Piaget [5] proposed that conceptions of moral wrongness and punishment change during childhood manifesting an outcome-to-intention shift, and a shift from retributivist expiation [40] to more mature notions that are based on reciprocal processes and compensation. More recently, it has been shown that conversations among peers may affect children's conceptions of punishment, diminishing the role of notions based on expiation [41]. The experiments reported here have little to say about how the explicit notions of punishment or fairness develop in preschoolers and older children [42]–[44]. However, the present results have one important implication for theories that are focused on when and how children acquire their explicit conceptions of punishment. Since an implicit encoding of antisocial actions as related to previous positive or negative actions may be present early in infancy, the representations employed in such processes may provide the foundations for the conceptions of punishment found in older children. A similar conclusion is also plausible for the development of the fairness concept employed in the evaluation of resource allocations.

Critics of the nativist perspectives on infants' sense of fairness emphasise the effect of experience (e.g. [29]). The crucial issue in assessing the viability of nativist models is not, however, whether experience has an effect on the development of infants' and children's evaluation of fairness: this is a well-established fact (e.g. [42]). Instead, the central issue is whether the biases and skills found in infants can be explained as the result of parsimonious domain-general mechanisms that allow them to detect statistical regularities in the environment. By lowering the age of participants to 10 months, the present results support the claim that infants' ability to attend to distributive actions as cues for agents' social evaluation does not emerge by linguistic processing or parental feedback, and suggest that the detection of environmental regularities plays a minor role, if any at all.
